# Developing an intervention to support lung cancer patients and their clinicians when considering systemic anti-cancer therapy: pact study

**DOI:** 10.1186/1745-6215-14-S1-P8

**Published:** 2013-11-29

**Authors:** Annmarie Nelson, Stephanie Sivell, Simon Noble, Anthony Byrne, Jason Lester

**Affiliations:** 1 Marie Curie Palliative Care Research Centre, Cardiff University School of Medicine, Cardiff, UK; 2 Velindre NHS Trust, Cardiff, UK

## Background

Lung cancer patients may inappropriately receive systemic anti-cancer therapy (SACT) when they are close to end of life, despite evidence that early palliative intervention can led to less aggressive care and longer survival [1]. The National Confidential Enquiry into Patient Outcome and Death made the recommendation that acceptance of treatment/treatment decision should be made by the patient after they have been fully informed of the risks and benefits [2].

## Aims

To identify the information and decision support needs of patients and to design an intervention to facilitate discussion of the risks and benefits of the available treatment options.

## Methods

Observations of interactions at MDT meetings and patient-clinician consultations (n=20-30) will identify how patients are allocated to treatment pathways, how treatment management options are presented to patients and the extent of patient involvement in treatment planning. Semi-structured face-to-face interviews with patients and clinicians will explore perceptions of the treatment management options, and attitudes and perceived involvement in treatment decisions. These data will be used to inform the design, development and subsequent evaluation of the intervention, including an appropriate theoretical basis.

## Results and conclusions

Data collection will commence in the autumn of 2013. We anticipate the data to provide an insight into why lung cancer patients may receive SACT when palliative care may perhaps be more appropriate. The resulting intervention will be made available to patients and clinicians to provide information and support when considering uptake of SACT for lung cancer.
